# Hetereogeneity in Neuronal Intrinsic Properties: A Possible Mechanism for Hub-Like Properties of the Rat Anterior Cingulate Cortex during Network Activity

**DOI:** 10.1523/ENEURO.0313-16.2017

**Published:** 2017-03-07

**Authors:** Natalie E. Adams, Jason S. Sherfey, Nancy J. Kopell, Miles A. Whittington, Fiona E.N. LeBeau

**Affiliations:** 1 Institute of Neuroscience, Medical School, Newcastle University, Newcastle-upon-Tyne, NE2 4HH, UK; 2 Department of Mathematics and Statistics, Boston University, Boston, MA 02215; 3 Center for Computational Neuroscience and Neural Technology, Boston University, Boston, MA 02215; 4 Department of Biology-F1, Hull York Medical School, York University, Heslington, YO10 5DD, UK

**Keywords:** anterior cingulate, dynamic routing, intrinsic properties, modeling, network oscillations

## Abstract

The anterior cingulate cortex (ACC) is vital for a range of brain functions requiring cognitive control and has highly divergent inputs and outputs, thus manifesting as a hub in connectomic analyses. Studies show diverse functional interactions within the ACC are associated with network oscillations in the β (20–30 Hz) and γ (30-80 Hz) frequency range. Oscillations permit dynamic routing of information within cortex, a function that depends on bandpass filter–like behavior to selectively respond to specific inputs. However, a putative hub region such as ACC needs to be able to combine inputs from multiple sources rather than select a single input at the expense of others. To address this potential functional dichotomy, we modeled local ACC network dynamics in the rat in vitro. Modal peak oscillation frequencies in the β- and γ-frequency band corresponded to GABA_A_ergic synaptic kinetics as seen in other regions; however, the intrinsic properties of ACC principal neurons were highly diverse. Computational modeling predicted that this neuronal response diversity broadened the bandwidth for filtering rhythmic inputs and supported combination—rather than selection—of different frequencies within the canonical γ and β electroencephalograph bands. These findings suggest that oscillating neuronal populations can support either response selection (routing) or combination, depending on the interplay between the kinetics of synaptic inhibition and the degree of heterogeneity of principal cell intrinsic conductances.

## Significance Statement

The anterior cingulate cortex (ACC) integrates diverse inputs and outputs to mediate higher cognitive functions. Experimental data demonstrated that fast network oscillations at both the β- and γ-frequency bands could be elicited *in vitro* in the rat ACC, and neurons exhibited a wide range of intrinsic properties. Computational modeling of the ACC network, constrained by the biological data, revealed that the frequency of network oscillation generated was dependent on the time course of inhibition. Neuronal response heterogeneity broadened the range of frequencies generated by the model network. In addition, with different frequency inputs to two neuronal assemblies, heterogeneity decreased competition and increased spike synchrony between the networks, thus conferring a combinatorial advantage to the network.

## Introduction

Anterior cingulate cortex (ACC) is a functionally distinct area of the prefrontal cortex (PFC) that in rats, primates, and humans is associated with a broad range of functions, including remote spatial memory (Teixeira et al., 2006; Wartman et al., 2014), attention and executive function (Kesner and Churchwell, 2011; Newman et al., 2014), adaptive control (Narayanan et al., 2013), error detection (Ito et al., 2003; Hyman et al., 2013), and reward-based decision-making (Walton et al., 2003; Hillman and Bilkey, 2010). Such diversity of function, collectively referred to as cognitive control (Shenhav et al., 2013), supports the idea that the ACC may act as a general action-outcome predictor (Hyman et al., 2013).

A wide range of inputs to ACC suggest that, from a connectomic point of view, this region can potentially monitor all modalities of primary sensory input, affective state, motor state, and associational processing (Hoover and Vertes, 2007; Park and Friston, 2013; Vogt and Paxinos, 2014). Equally diverse outputs from ACC (Gabbott et al., 2005) would allow this area to function as a higher-order hub region, vital for multimodal integration (Bassett et al., 2008).

A dynamic view of brain connectivity reveals a rich variation in the interplay between one brain region and others that change over time scales of less than tens of milliseconds (Kopell et al., 2014). Functional connectivity, with quantifiable behavioral consequences, involves specific phase relationships between activity in different areas organized over a spectral range including β (20–30 Hz) and γ (30–80 Hz) frequency electroencephalograph (EEG) bands (Bastos et al., 2015). Within this frequency range, local neuronal networks can effectively select which inputs to respond to on the basis of the relative peak frequency of concurrent inputs—with faster frequencies taking precedence (Cannon et al., 2014). This selection of inputs can lead to routing of information based on the relative strength of the oscillations present in the inputs (Kopell et al., 2010; Akam and Kullmann, 2010, 2014).

Network oscillations in the β- and γ-frequency range in PFC subregions, including ACC, are associated with many cognitive functions (Buschman et al., 2012; Brincat and Miller, 2015). This activity depends on local network interactions between excitatory principal cells and fast-spiking, parvalbumin-containing (PV) interneurons (e.g., Fisahn et al., 1998; Whittington et al., 2011). Computational studies have predicted that the input selection and routing behaviors of γ and β rhythms are, at least in part, manifested through the establishment of bandpass-filter behavior of local networks afforded by the kinetics of the synaptic inhibition provided by these PV neurons (Akam and Kullmann, 2010; Cannon et al., 2014). In contrast, it can be argued that the function of hub-like regions such as ACC should depend more on a combinatorial processing of inputs, with selection and routing processes more appropriate for regions critical for contextual disambiguation (Phillips et al., 2010).

Using *in vitro* recordings in rat ACC, we investigated the network properties of β- and γ-frequency oscillations generated locally and the diversity of intrinsic cellular properties of ACC principal cells. The data demonstrated that the generation of both β- and γ-frequency oscillations was critically dependent on the kinetics of fast synaptic inhibition, as widely reported in other brain regions (Whittington et al., 2011). Here we show that both passive and active intrinsic principal cell properties exhibited a great deal of heterogeneity. Computational studies, constrained by this biological data, showed that in response to rhythmic (sinusoidal) input, the kinetics of slow (13 ms) and fast (5 ms) synaptic inhibition decay times determined the center frequency of the bandpass-filtering properties of the ACC network to within either the β- or γ-frequency range, respectively. However, modeling the observed heterogeneity in the intrinsic properties of ACC neurons broadened the filter bandwidth at both β and γ frequencies. The main consequence of the broader filter bandwidths was to bias the local network behavior away from input selection and toward a more combinatorial behavior, consistent with the proposed hub-like function of ACC.

## Methods

### Slice preparation and solutions

Coronal slices (450 µm thick) containing the caudal regions of ACC (Cg1 and Cg2) were prepared from 2- to 3-month-old male Lister hooded rats. Rats were anesthetized with inhaled isoflurane, followed by an intramuscular injection of ketamine (100 mg/kg; Fort Dodge Animal Health) and xylazine (10 mg/kg; Animalcare). When all responses to noxious stimuli, such as pedal withdrawal reflex, had terminated, the animals were intracardially perfused with ∼50 ml of modified artificial CSF (ACSF) composed of (in mm) 252 sucrose, 3.0 KCl, 1.25 NaH_2_PO_4_, 24 NaHCO_3_, 2.0 MgSO_4_, 2.0 CaCl_2_, and 10 glucose. All procedures were in accordance with the UK Animals (Scientific Procedures) Act of 1986 and the European Union Directive 2010/63/EU.

After brain removal, 450-μm-thick coronal PFC slices were cut using a Leica VT1000S vibratome. Slices were then trimmed and transferred to a holding chamber at room temperature for ∼1 h, which allowed washout of all anesthetic agents, before being transferred to a recording chamber, where they were maintained at ∼29–31°C at the interface between normal ACSF (where sucrose was replaced with 126 mm NaCl, and MgSO_4_ and CaCl_2_ were reduced to 1.2 and 1.76 mm, respectively) and humidified 95% O_2_/5% CO_2_. Drugs used were as follows: kainic acid (800 nm; Sigma-Aldrich); the AMPA/kainate receptor antagonist 2,3-dioxo-6-nitro-1,2,3,4-tetrahydrobenzo[f]quinoxaline-7-sulfonamide (NBQX; 10 µm); the NMDA receptor antagonist d-(-)-2-amino-5-phosphonopentanoic acid (D-AP5; 50 µm); and the GABA_B_ receptor antagonist 3-aminopropyl-diethoxy-methyl-phosphinic acid CGP 35348 (5 µm).

### Recording techniques

Extracellular recordings were performed with ACSF-filled glass microelectrodes (resistance <5 MΩ). Only one extracellular electrode was positioned in each slice, which was moved across the laminae until a clearly detectable oscillation was recorded. Intracellular recordings used glass microelectrodes (resistance 80–150 MΩ) filled with potassium acetate (2 m). To assess intrinsic membrane properties, ACC cells were recorded with antagonists of glutamatergic transmission including NBQX (10 µm), D-AP5 (50 µm), and CGP 35348 (5 µm) in the ACSF. Data were recorded via an Axoclamp amplifier (Molecular Devices), using an InstruTECH ITC-16 (HEKA Electronic, Digitimer) after live mains noise was removed using a Humbug (Quest Scientific, Digitimer) and preamplification and bandpass filtering between 1.5 and 300 Hz (Neurolog, Digitimer). Data were sampled at 5 kHz.

### Data analysis

All time-series analyses were performed in MATLAB (MathWorks). Power spectra for all local field potential (LFP) and intracellular recordings were created using Welch’s estimate on traces between 20 s and 1 min in length, using a window length of 5 s and a 50% window overlap. During postprocessing, the LFP data were processed up to only a frequency of 80 Hz.

### Postsynaptic potentials

**Figure 1.**
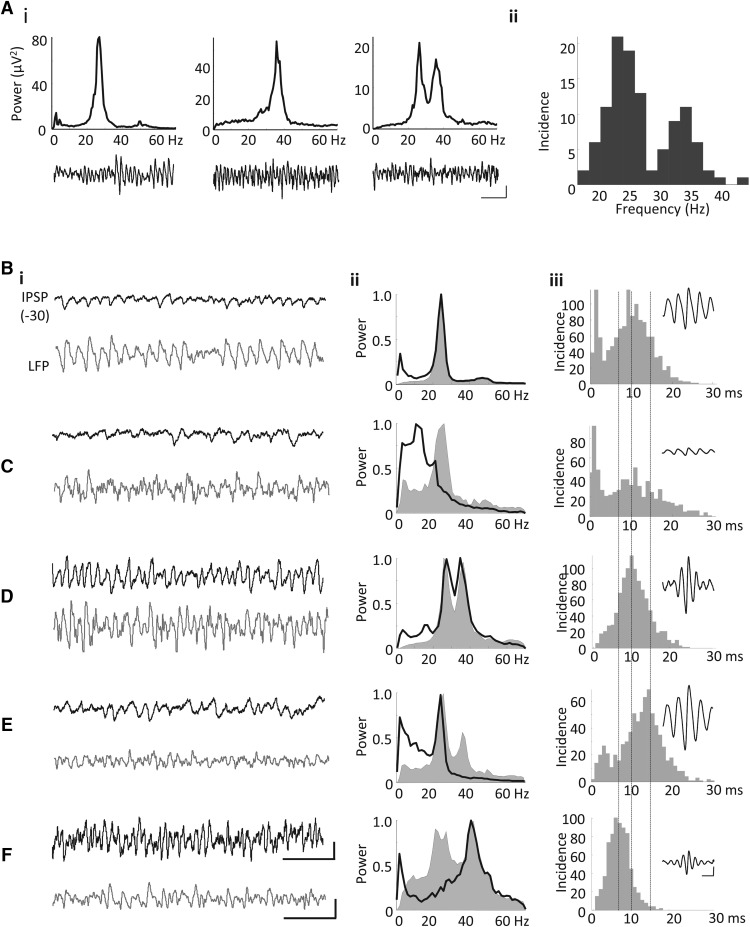
IPSPs suggest dual inhibitory inputs in ACC cells during γ/β network oscillations. ***Ai***, Example power spectra and traces of KA-evoked oscillations showing representative local field potential oscillations from three different slices consisting of purely β rhythms (left), purely γ rhythms (middle), and a dual-frequency oscillation (right); scale bar 50 μV, 200 ms. ***Aii***, Incidence plot for the modal peak frequencies in spectra from 109 ACC slices showing a clear bimodal average spectrum. ***Bi***, ***Bii***, Example IPSP traces (scale bar 5 mV, 200 ms) and simultaneously recorded LFP (scale bar 50 μV, 200 ms; ***i***) with corresponding normalized power spectra (***ii***) showing the LFP power spectrum (shaded) and overlaid IPSP power spectrum (black line). ***Biii***, Distribution of IPSP decay times. Inset shows IPSP-triggered averages of the LFP. ***B***, Field oscillation and IPSP at β frequency. ***C***, Field oscillations at β frequency but IPSPs at ∼12 Hz. ***D***, Mixed γ/β frequency field oscillation and IPSPs. ***E***, Mixed γ/β field oscillations but IPSPs only at β frequency. ***F***, Mixed γ/β field oscillations but IPSPs only at γ frequency.

Inhibitory postsynaptic potentials (IPSPs) were recorded with cells held at a membrane potential of –30 mV (Fig. 1). IPSPs were analyzed using a custom-made Matlab script and were included only if the amplitude exceeded 0.5 mV. IPSP amplitude was calculated as the voltage difference between the trough of the IPSP and the preceding peak (start of the IPSP). Decay times of IPSPs were taken at 63% of the peak voltage deflection. Spike-triggered averaging was performed by taking the mean of all spiking events above a threshold of –40 mV with the spike peak forming the center of the average and including 50 ms before and 200 ms after the peak.

### ACC cell intrinsic properties

**Figure 2.**
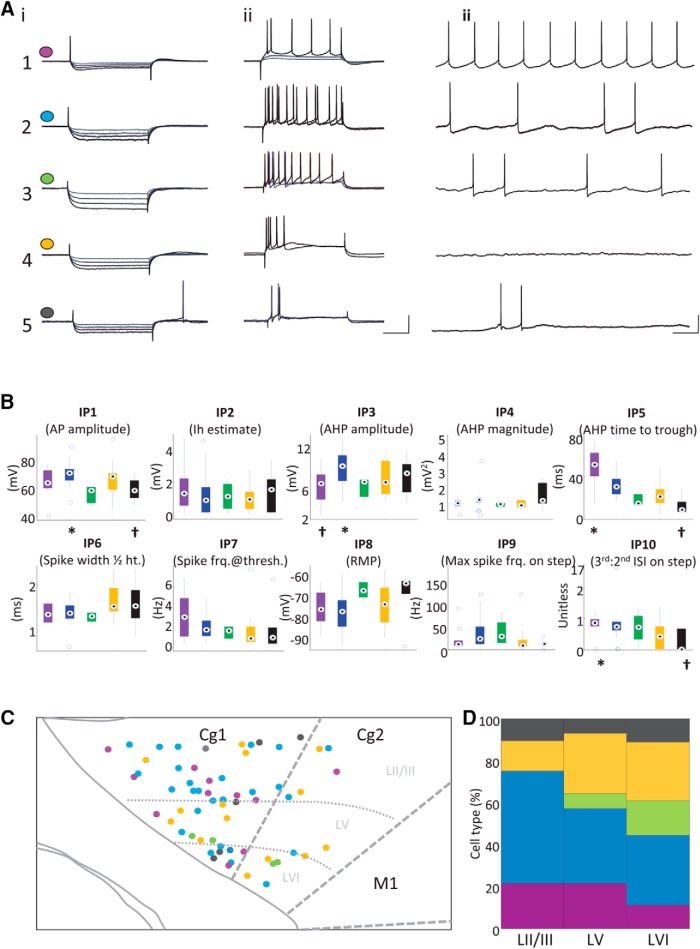
Manual classification and laminar distribution of cells in ACC. ***A***, Each row shows the electrophysiological response properties of one example cell from groups 1–5: 400-ms hyperpolarizing steps at 0.1–0.4 nA (***i***); 400-ms depolarizing steps at 0.3–0.4 nA (***ii***; gray to black = smaller to larger current step; scale bar 20 mV, 100 ms). ***Aiii***, Tonic activity at spike threshold (scale bar 20 mV, 200 ms). ***B***, Values (medians and IQRs) for 10 IPs plotted for each manually selected group 1–5 (colors as in ***A***). ^†^Significantly different from * (one-way ANOVA, *p* < 0.05). Central circle, median values; blue circles, outliers. ***C***, Schematic diagram of ACC (Cg1 and Cg2) with dots showing the location of cells found with different response properties. The color of each dot corresponds to cells from groups 1–5 recorded at each location. ***D***, Plot shows the laminar distribution profile as a percentage of total cells in groups 1–5.

Numerical values for intrinsic parameters (IPs) were collated for each cell (Fig. 2). The chosen IPs were as follows: IP1, action potential (AP) amplitude (mV); IP2, *I_h_* (cAMP-dependent hyperpolarization-activated current) estimate (the amplitude of the sag potential from the steady state of –200 nA step injection and the subsequent return to rest; mV); IP3, afterhyperpolarization (AHP) amplitude (mV); IP4, AHP magnitude (voltage integrated over 200 ms post-AP; mV); IP5, AHP time to maximal deflection (ms); IP6, spike width at half-height (ms); IP7, spontaneous spike rate at threshold (Hz); IP8, resting membrane potential (rmp; mV); IP9, initial spike frequency (time between first and second spikes) on step depolarization (Hz); IP10, ratio of third to second interstimulus interval on step depolarization-induced spike train (used as a measure of spike frequency adaptation; unitless). After within-parameter normalization, the normalized cross-correlation coefficients between pairs of parameters were found. IPs that were correlated (or anti-correlated) above an absolute value of ±0.33 were considered significantly correlated and were discarded. The variance explained in the remaining IP dataset (IP3, IP5–IP10) was calculated for each property.

### Cell clustering

**Figure 3.**
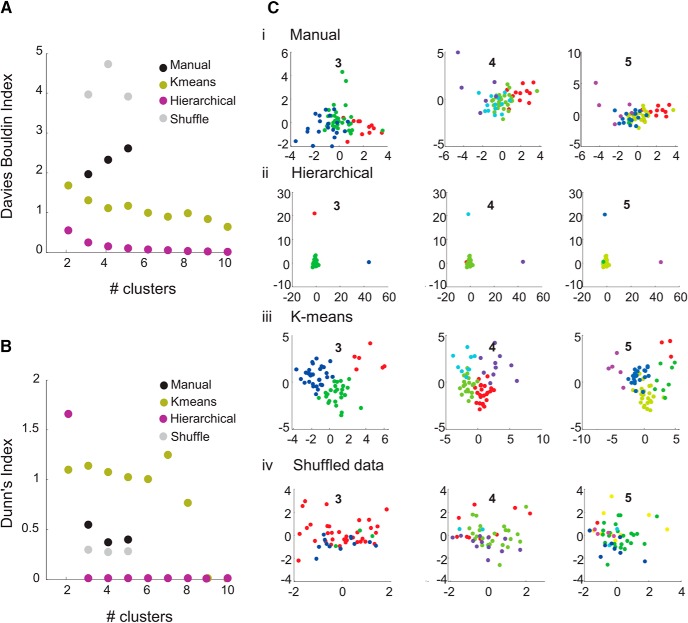
Objective clustering analysis does not identify distinct clusters. Validity indices Davies–Bouldin (***A***) and Dunn’s (***B***) for the three clustering methods attempted; manual (black circles), *k*-means (olive circles), hierarchical (purple circles), and shuffled data (gray circles) for a range of cluster numbers (2–10 clusters). ***C***, 2D canonical variable plots (unitless) from the multivariate analysis for the manual (***i***), hierarchical (***ii***), *k*-means (***iii***), and shuffled (***iv***) clusters when assuming three, four, and five clusters. Each plot shows the cells of each cluster as an arbitrary color.

Clustering was performed using *k*-means and hierarchical methods (Fig. 3). One-way ANOVA was used to compare parameter distributions across the clusters. After within-parameter normalization, the normalized cross-correlation coefficients between pairs of parameters were found, producing a correlation matrix of all pairs. Variables that were correlated (or anti-correlated) above an absolute value of ±0.33 were classed as significantly correlated variables and were discarded. Multivariate ANOVA analyses of the remaining parameters were performed on all pairs of clusters to determine whether cells could be clustered according to individual or combined parameters. Hierarchical clustering used Ward’s minimum variance criterion. The effectiveness of clustering was tested for 2–10 clusters using the Davies–Bouldin index, which measures the ratio of intracluster scatter to intercluster separation, and Dunn’s Index, which similarly assesses intracluster distance versus intercluster distance. Maximization of the Davies–Bouldin index and minimization of Dunn’s Index provide internal measures of the effectiveness of the clustering (Davies and Bouldin, 1979; Maulik and Bandyopadhyay, 2002). Data were shuffled by reordering the values for each intrinsic property via a random permutation in Matlab.

### Statistical analysis

Statistical analyses were performed in Sigmaplot 11.0 (Systat Software), and data are presented as median and 25%–75% interquartile range (IQR). Significance chosen was *p* < 0.05.

### Computational modeling

#### Pyramidal cell model

**Figure 4.**
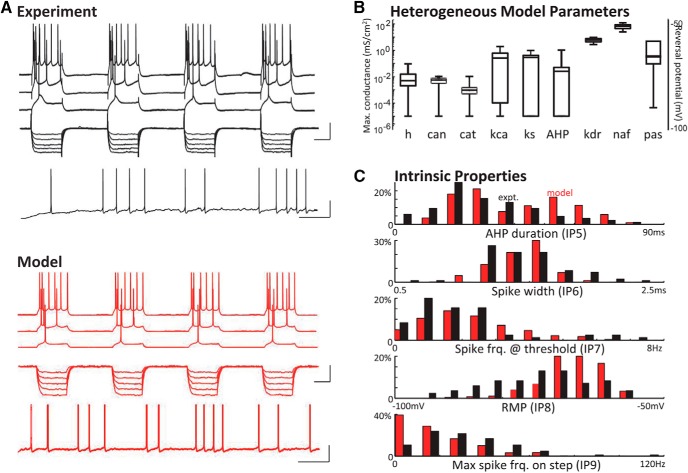
Heterogeneous biophysical models reproduce the range of experimental intrinsic properties. ***A***, Example of experiment and model cell responses to depolarizing current steps (top) and hyperpolarizing current steps (middle) and firing rate at rmp (bottom). Scale bars 20 mV/100 ms and 20 mV/2 s. ***B***, Ion channel conductance ranges (10^−6^ to 10^2^) used in ACC cell model. Values are based on model fits to experimental IP ranges. Ion channel key: h, HCN channel; can, high-threshold (N-type) calcium channel; cat, low-threshold (T-type) calcium channel; kca, calcium-dependent potassium channel; ks, slow (M-type) potassium channel; AHP, slow afterhyperpolarizing potassium channel; kdr, fast delayed rectifier potassium channel; naf, fast sodium channel; pas, passive leak channel. ***C***, Model and experimental IP distributions for the 5 IPs (IP5–9) that explained most of the variance.

We developed a single-compartment pyramidal cell model capable of producing the range of intrinsic membrane properties observed in ACC (Fig. 4). The conductance-based model had a membrane potential V (mV) governed by

$$C_{m}\frac{dV}{dt}=I_{inj}\left(t\right)-\sum I_{int}+\eta ,$$

where *t* is time (ms), *C_m_* = 1 µF/cm^2^ is the membrane capacitance, η = 0.01ε is Gaussian noise with ε ∼ *N*(0,1) and amplitude tuned to match baseline fluctuations in membrane potential, *I_inj_*(*t*) is an injected current (µA/cm^2^) simulating an *in vitro* experimental protocol, and *I_int_* denotes intrinsic membrane currents (µA/cm^2^) produced by ionic conductances with Hodgkin–Huxley-like channel kinetics from published cell models: *I_KDR_*, *I_NaF_*, *I_KS_*, and *I_NaP_* from Durstewitz and Seamans (2002) for spike generation and adaptation; *I_CaN_*, *I_CaT_*, *I_KCa_*, and *I_H_* from Papoutsi et al. (2013) for calcium-dependent adaptation, slow afterhyperpolarization, and hyperpolarization-induced voltage sag; and *I_AHP_* from Yamada et al. (1998) (see legend of Fig. 4 for more details). Ionic currents from Durstewitz and Seamans (2002) and Papoutsi et al. (2013) were chosen because their kinetics were constrained by experimental data from rat medial PFC. Each active current was modeled as $I_{int}=g_{int}m^{p}h^{q}\left(V-E_{int}\right)$, where maximal conductance *g_int_* (mS/cm^2^), reversal potential *E_int_* (mV), *p*, *q*, and the kinetics of activation gate *m* and inactivation gate *h* were as published unless otherwise specified. All cells had a passive leak current $I_{L}=g_{L}\left(V-E_{L}\right)$ with conductance *g_L_* = 0.04 mS/cm^2^ and reversal potential *E_L_* (mV).

The *in vitro* cell characterization experiment was simulated by a current injection, *I_inj_*(*t*), delivering a series of hyperpolarizing and depolarizing pulses, followed by a ramp to spike threshold, then constant depolarization (compare Fig. 4*A*). Model IPs were calculated from the simulated data using the same analysis applied to the experimental recordings. A set of cell models capturing the diversity observed in ACC was obtained by manually varying biophysical parameters and comparing model IPs to the aggregate (all cells, all layers) experimental distributions (Fig. 4*C*). Specifically, *E_L_* and maximal values for eight active conductances (*g_KDR_*, *g_NaF_*, *g_KS_*, *g_CaN_*, *g_CaT_*, *g_KCa_*, *g_H_*, *g_AHP_*) were varied across simulations to find a set of models with IPs spanning the ACC distributions for the five most discriminative IPs from the experimental IP data analysis (IP5–IP9, accounting for 85% of the total variance in ACC IPs; see Fig. 4*C*). Parameter space was explored in two steps. First, each maximal conductance was logarithmically varied 1 × 10^–6^ to 1 × 10^2^ to determine the scales over which realistic IPs could be observed. Next, hypercube subspaces were explored around the identified scales for maximal conductance and an *E_L_* range spanning the recorded rmp values. Only parameter sets producing IPs within the experimental ranges were considered viable models of the pyramidal cells recorded *in vitro* in the presence of synaptic blockers. This procedure resulted in 2810 viable cell models of >100,000 simulated models.

All viable cell models had biophysical parameters yielding intrinsic electrophysiological properties within the ranges observed experimentally. Across the set of viable models, each biophysical parameter had a distribution of values (Fig. 4*C*) and tended to covary with other biophysical parameters to some degree. We defined a homogeneous assembly of cells as a population of equivalent cell models with biophysical parameters set to medians computed across the full set of viable models; all cells belonging to a given assembly received similar inputs (see details below). Independent realizations of heterogeneous assemblies were generated by drawing biophysical parameters from a multivariate normal distribution using the covariance matrix, including all pairs of biophysical parameters, computed across the full set of 2810 viable models.

#### Biophysical network model

We developed a computational representation of a generic ACC network including single-compartment excitatory (E) pyramidal cells and inhibitory (I) interneurons. E-cells were modeled as previously described with the addition of synaptic inputs and exclusion of the injected current:

$$C_{m}\frac{dV}{dt}=-I_{ex}\left(t,V\right)-\sum I_{int}-\sum I_{syn},$$

where *I_ex_*(*t,V*) is an excitatory current (µA/cm^2^) reflecting inputs from external sources and *I_syn_* denotes synaptic currents (µA/cm^2^) driven by other E- and I-cells in the network. I-cells were modeled using the fast-spiking (FS) Wang–Buzsáki interneuron model (Wang and Buzsáki, 1996). A more computationally demanding FS I-cell model based on PFC data (Durstewitz and Seamans, 2002) produced qualitatively similar results.

**Figure 5.**
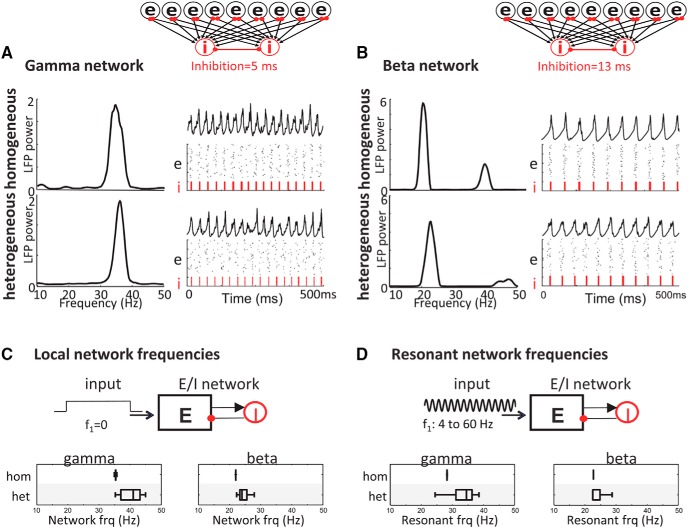
Cell diversity broadens intrinsic (local) oscillations and network tuning in ACC model. ***A***, ***B***, Network models were constructed by coupling the heterogeneous E-cell population to I-cells with time constants of inhibition based on the IPSP durations observed in cells rhythmic with the network β or γ rhythm in the LFP. The resulting E/I networks with fast (5 ms) and slow (13 ms) inhibition produced γ-frequency (***A***) and β-frequency (***B***) network oscillations whether the E-cell population had homogeneous or heterogeneous IPs. ***C***, Effect of cell diversity on the intrinsic (local) frequency of network oscillations: Poisson noise input was applied to different 80-cell subsets of network E-cells on different realizations. Box plots show range of network frequencies for homogeneous and heterogeneous networks with different inhibition time constants at β and γ frequencies. ***D***, Effect of cell diversity on network tuning (resonant frequency): a sinusoidal input was applied to different subsets of E-cells on different realizations, independently for each input frequency 4–60 Hz (in 2-Hz steps). Box plots show range of resonant frequencies of the homogeneous and heterogeneous networks.

**Figure 6.**
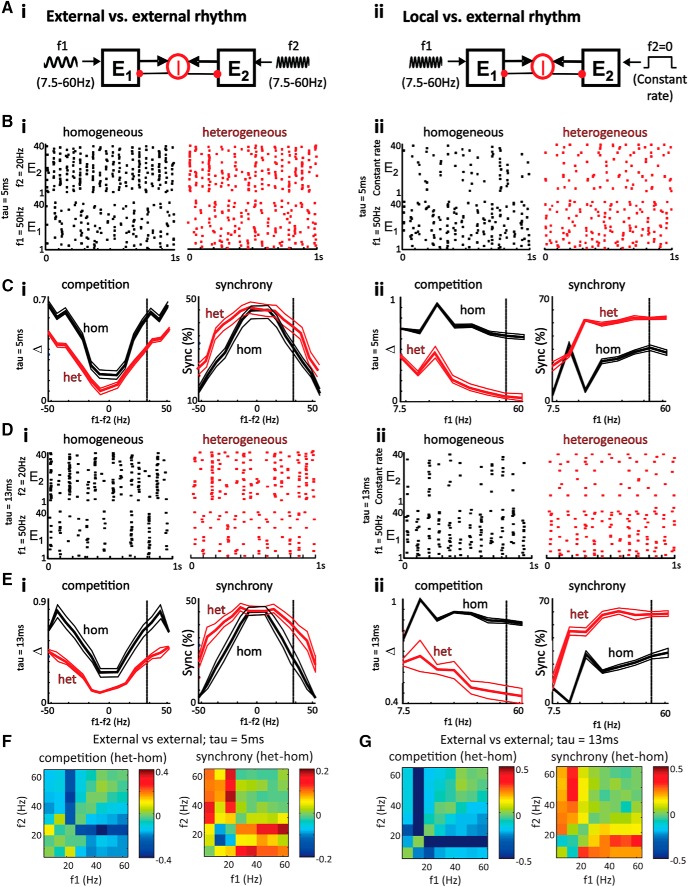
Heterogeneity increases synchrony and decreases competition between cell assemblies. ***Ai***, Model schematic showing two excitatory assemblies, E1 and E2, receiving rhythmic AMPAergic inputs with equal spike counts and time-varying Poisson rates modulated at frequencies *f*_1_ and *f*_2_, respectively. The assemblies compete through a shared pool of inhibitory interneurons (I-cells), and τ*_I_* = 5 ms and τ*_I_* = 13 ms were used for these results. ***Bi***, For homogeneous assemblies (left) driven by external rhythms, the assembly with a more resonant input (e.g., 20 Hz) suppresses spiking in the assembly driven by a less resonant input (e.g., 50 Hz). Heterogeneity of cell intrinsic properties decreases this competition (right) and increases synchrony between the two assemblies (i.e., the fraction of 10-ms bins with spiking in both E1 and E2). ***Ci***, Heterogeneity decreases competition (Δ) across all pairs of input frequencies and increases synchrony for inputs separated by >30 Hz. Solid lines represent the *f*_1_ – *f*_2_ shown in the above raster plots. ***Di***, ***Ei***, Similar raster plots and plots of competition and synchrony for τ*_I_* = 13 ms. Again, heterogeneity decreases competition across all pairs of input frequencies and increases synchrony for inputs separated by >30 Hz. ***Aii***, Model schematic showing two assemblies, E1 and E2, receiving external rhythmic and background noise inputs, respectively, with the latter driving a local rhythm at the natural frequency of E2 (as in Fig. 5*A*). ***Bii***, The less resonant input from ***Bi*** strongly suppresses an assembly driven by a nonrhythmic Poisson input with equal spike count and constant rate. Heterogeneity decreases competition and increases synchrony. ***Cii***, Heterogeneity again decreases competition for all input frequencies and increases synchrony for frequencies >20 Hz. ***Dii***, ***Eii***, Similar raster plots and plots of competition and synchrony for τ*_I_* = 13 ms; again, heterogeneity decreases competition and increases synchrony in a very similar manner to τ*_I_* = 5 ms. Solid lines represent the *f*_1_ shown in the above raster plots. ***F***, Plots show differences (heterogeneous – homogeneous) in mean competition and synchrony for τ = 5 ms plotted as *f*_1_ against *f*_2_ on separate axes. ***G***, Same as ***F*** except τ = 13 ms.

All networks consisted of 80 E-cells split into one or two assemblies coupled reciprocally to a shared pool of 20 I-cells (see model architecture in Figs. 5*A*, 5*B*, and 6*A* ). E-cells provided excitation to all I-cells, mediated by α-amino-3-hydroxy-5-methyl-4-isoxazolepropionic acid (AMPA) currents. I-cells in turn provided inhibitory inputs γ-aminobutyric acid (GABA_A_) currents to all E-cells and I-cells. AMPA currents were modeled as

$$I_{AMPA}=g_{AMPA}s\left(V-E_{AMPA}\right),$$

where *V* is the postsynaptic membrane voltage, *g_AMPA_* is the maximal synaptic conductance, *s* is a synaptic gating variable, and $E_{AMPA}=0\text{ mV}$ is the synaptic reversal potential. Synaptic gating was modeled by

$$\frac{ds}{dt}=H\left(V_{pre}\right)\frac{1-s}{\tau_{r}}-\frac{s}{\tau_{d}},$$

where *V_pre_* is the presynaptic membrane voltage, τ*_r_* = 0.4 ms and τ*_d_* = 2 ms are time constants for neurotransmitter release and decay, respectively, and H(V)=1+tanh(V/10) is a sigmoidal approximation to the Heaviside step function. GABA_A_ currents are modeled in the same way with $E_{GABA}=-75\text{ mV}$ and variable τ*_d_* = τ*_I_* (5 or 13 ms, reflecting inhibition from different interneuron classes). Maximum synaptic conductances for E-cells, GABA_A_ (0.1 mS/cm^2^); for I-cells, AMPA (1 mS/cm^2^) and GABA_A_ (1 mS/cm^2^).

Excitatory/inhibitory (E/I) networks with one E-cell assembly were simulated to probe the natural and resonant frequencies of ACC networks with and without E-cell heterogeneity (Fig. 5). In these simulations, the input was a gated channel with excitatory AMPA current, $I_{ex}=g_{ex}s_{ex}\left(V-E_{ex}\right)$, where *E_ex_* is the synaptic reversal potential and *s_ex_* is a postsynaptic gating variable that integrates independent Poisson spike trains with time-varying rate function λ(*t*). In different simulations, λ(*t*) = (background activity) was used to probe natural frequencies λ(t)=r[1 + sin(2 πft)]/2 (rhythmic input) was used to probe resonant frequencies. Input spike trains represent background noise or rhythmic population activity originating from an arbitrary source network and are integrated in the synaptic gate *s_ex_* with exponential AMPAergic decay. *r* was tuned during the former simulations to produce firing rates observed in the *in vitro* experiments with kainate-induced network oscillations. In the latter simulations, *f* was varied from 4 to 60 Hz (2-Hz steps) across simulations. All single-assembly simulations were repeated for τ*_I_* = 5 and 13 ms based on experimentally observed IPSPs in the γ- and β-coherent cells, respectively. Parameters were set to *E_ex_* = 0, *g_ex_* = 0.001, and *r* = 4500 Hz.

E/I networks with two identical E-cell assemblies, E1 and E2, were simulated to probe the effects of heterogeneity on synchrony (integration) and competition between assemblies receiving noise or rhythmic inputs at different frequencies, *f*_1_ and *f*_2_, respectively (Fig. 6). In these simulations, the excitatory input was a gated AMPAergic response to a Poisson spike train as before. However, assemblies E1 and E2 received inputs from distinct source networks. Source network frequencies (*f*_1_, *f*_2_) were varied over a grid ranging from 7.5 to 60 Hz (7.5-Hz steps) across simulations, while the number of spikes delivered to each assembly was fixed. τ*_I_* = 5 ms and τ*_I_* = 13 ms were both used for the results reported below.

The diversity of cell intrinsic properties observed in ACC pyramidal cells was incorporated into heterogeneous E-cell assemblies as previously detailed. For a given simulation with two assemblies, the same cell models were used for both assemblies to ensure that differences in their activities resulted from differences in their inputs and not differences in their cell properties.

All models were implemented in Matlab using the DynaSim toolbox (github.com/DynaSim) and are publicly available online at: github.com/jsherfey/ACd_model. Numerical integration was performed using a fourth-order Runge–Kutta method with a fixed time step of 0.01 ms. One- and two-assembly network simulations were run for 2000 and 5000 ms, respectively, and the first 500 ms was excluded from subsequent analysis. All network simulations were repeated 10 times.

#### Model analysis

*Analysis of model networks with one assembly*. The natural frequency of a network is the frequency of rhythmic population activity that emerges naturally given background activity. The natural frequency was identified as the frequency with peak power in Welch’s spectrum of the mean E-cell voltage (simulated LFP) given an external input with constant *g_ex_*. The resonant frequency of a network is the frequency of a rhythmic input for which the network exhibits maximal spiking. The resonant frequency was identified as the input frequency producing the maximum number of spikes in the E-cell assembly given an external input with sinusoidal *g_ex_*.

*Analysis of model networks with two assemblies*. Two E-cell assemblies coupled to a shared pool of I-cells may differ in their amount of spiking (i.e., they may compete) or exhibit synchronous spiking to varying degrees (i.e., they may or may not support integration). The degree of competition between two assemblies, E1 and E2, was quantified by

$$\Delta =\frac{\left|N_{1}-N_{2}\right|}{N_{\max}},$$

where *N*_1_ is the number of spikes in assembly E1, *N*_2_ is the number of spikes in assembly E2, and *N*_max_ is the number of spikes in the more active assembly. Δ indicates how much more active a dominant assembly is compared with a less active assembly; it varies between 0 (equal activity levels) and 1 (total suppression of the nondominant assembly). The degree of spike synchrony between two assemblies was quantified using the percentage of 10-ms time bins for which spiking occurred in both assemblies. Competition and synchrony were compared between homogeneous and heterogeneous networks using a two-sample *t* test and were considered significant if *p* < 0.05.

## Results

### Kainate-evoked network oscillations in ACC

Glutamatergic excitation via bath application of the kainate receptor agonist kainic acid (KA; 800 nM) was the sole pharmacological manipulation necessary to produce a range of network oscillatory activity. Example power spectra with the associated LFP traces from three different experiments showed that the ACC oscillations could consist of either a single peak at β frequencies (*n* = 81/109; 74%), a single peak at γ frequencies (*n* = 16/109; 15%), or dual peaks at both β and γ frequencies (*n* = 12/109; 11%), (Fig. 1*Ai*). Oscillations at γ and β frequency could be observed in both deep and superficial layers. LFP recordings from all layers of ACC were combined, and the frequency of the oscillation evoked varied from 18 to 44 Hz (*n* = 109 slices) but resulted in a bimodal distribution with peaks at β frequencies (∼24 Hz) and γ frequencies (∼34 Hz; Fig. 1*Aii*).

### Local network inhibition

IPSPs were recorded during KA-evoked field oscillations from morphologically unidentified cells in ACC (*n* = 10), and a variety of different IPSP properties were observed. When a β-frequency oscillation was recorded in the LFP, the IPSPs recorded intracellularly were either rhythmic with the recorded LFP (Fig. 1*Bi*) or nonrhythmic with the concurrently recorded LFP (Fig. 1*Ci*). If the IPSPs were nonrhythmic at the LFP frequency (Fig. 1*Ci*), they still exhibited rhythmicity, but with a peak power below ∼12 Hz. When dual β-γ oscillations were recorded in the LFP, the IPSPs were either rhythmic at both frequencies (Fig. 1*Di*) or at only β (Fig. 1*Ei*) or γ (Fig. 1*Fi*) frequency.

The decay times for the IPSPs that were rhythmic with the β-frequency field oscillation were slower (modal peak 15 ± 3.5 ms; Fig. 1*Bi*) than IPSPs found to be rhythmic with the γ-frequency field oscillation (modal peak 6 ± 1.8 ms; Fig. 1*Fi*). In total, 8 of 10 cells were rhythmic with the field at either β or γ frequency. Overall, the results demonstrated that network inhibitory inputs mostly correlated with the bimodal nature of peak spectral frequencies seen in local field potentials such that IPSPs were largely either at β or γ frequency only. A correlation between field and IPSP frequency has been reported many times for network oscillations in primary sensory and polymodal association areas, reflecting the critical role of synaptic inhibition in shaping fast network dynamics (Whittington et al., 2011).

The above results revealed nothing unique in the profile of synaptic inhibition in ACC that could reflect the proposed hub-specific dynamic behavior that we predicted should be present in this region. We therefore switched our attention to examining the intrinsic principal neuronal properties that are known to be diverse in both rat (Yang et al., 1996; van Aerde and Feldmeyer, 2015) and primate (Ardid et al., 2015) PFC.

### ACC intrinsic cell properties

We recorded from 61 cells in the ACC in the presence of excitatory transmitter blockers (see Methods) and found a wide variety of IPs, as has been previously reported in the prelimbic and infralimbic regions of the PFC (Yang et al., 1996; Dembrow et al., 2010; Gee et al., 2012; van Aerde and Feldmeyer, 2015; Glykos et al., 2015).

Initially, cells were characterized manually by segregating cells according to their AHP shape and firing characteristics from step and tonic depolarizations. Fig. 2*A* illustrates the variety of electrophysiological characteristics, organized into manually created categories (groups 1–5). The presence of a distinct fast AHP was evident in some cells, either with or without an afterdepolarization (ADP) potential. Tonic injected current at the threshold for spiking showed that some cells fired continually, whereas others fired less frequently, with a low frequency intrinsic subthreshold oscillation (ISO) evident between spikes. Other cells did not fire at all on depolarization or required rapid acceleration of depolarizing current to produce any spikes. Some cells had very little spike accommodation, whereas others had very fast adapting properties. The five manually classified groups were defined as follows (Fig. 2*A*): group 1 (11 of 61, 18% of cells) had an AHP duration (∼200 ms), usually with a small ADP, little spike adaptation, and a spiking frequency of ∼5 Hz at threshold. Group 2 (27 of 61, 44% of cells) had similar properties to group 1 with respect to spike adaptation, but the AHP had a more variable duration (∼100–500 ms), and firing rates at threshold were less regular. Group 3 (3 of 61, 5% of cells) had a sharp AHP, clear spike adaptation, and irregular firing rates at threshold. Group 4 (14 of 61, 23% of cells) had very strong spike adaptation, rounded, short AHPs, and little or no firing after the initial spikes at threshold. Group 5 (6 of 61, 10% of cells) always had a characteristic fast AHP, interrupted by an ADP, then a long AHP, and exhibited strong spike adaptation. Without morphologically identifying cells, we could not determine whether any of these groups corresponded to classes of interneurons, rather than putative pyramidal cells, but no distinct fast-spiking (FS) interneurons were recorded in this study.

In all cells, we measured 10 intrinsic properties (IP1–10; Fig. 2*B*) as described in Materials and Methods. Each of these IPs reflects the presence and magnitude of intrinsic conductances that are known to influence neuronal resonance (input-filtering) properties (see Discussion). The IPs of all cells were compared across each of the manually selected groups (groups 1–5), but very few significant differences were found, and those that were identified did not show any obvious pattern (Fig. 2*B*). In addition, the distribution of cells in group 1–5 across the laminar structure of ACC was diffuse, as most cell types could be found in all layers (Fig. 2*C*, *D*). These data demonstrate that in the ACC, different cell classes could not be defined by either a unique expression of ion channel properties or laminar position.

To assess whether ACC cells could be separated into discrete clusters, both hierarchical and *k*-means clustering were used in an attempt to segregate the cells, assuming 2 to 10 clusters. The Davies–Bouldin index and Dunn’s index for a range of clusters from 2 to 10 for all clustering methods used in this study are plotted (Fig. 3*A*, *B*). In the Davies–Bouldin plot (Fig. 3*A*), the lower the index value, the better the cluster separation. Using Dunn’s Index (Fig. 3*B*), the higher the index value, the better the cluster separation. *k*-means performed the best overall at various cluster sizes, followed by manual clustering; shuffled data gave the worst performance. The hierarchical cluster analysis performed best at the two-cluster level, but other values on, or close to, the zero line suggest that this analysis failed at higher cluster numbers. However, although these data show that our manually selected clusters performed better than the shuffled data, the clusters were still not clearly separated by any method used here. All clustering of cells found using the different methods are shown for three, four, and five assumed clusters on 2D plots (Fig. 3*C*) of the first two canonical variables from the MANOVA analysis. For *k*-means clustering, at the three-cluster level (Fig. 3*Ciii*, left), all three clusters were significantly different from each other in the first canonical variable dimension (*x*-axis; *p* < 0.05). However, although the *k*-means method performed optimally of the chosen methods in terms of the validity tests (Dunn’s and Davies–Bouldin) as described above, two of the clusters (green and blue) can be seen to lie along a continuum, with no clear space separating them. In addition, relating these clusters individually back to the original electrophysiological characteristics yielded few significant differences, as with the manually selected clusters. At the three-cluster level using *k*-means, one cluster was separable (*p* < 0.05) in terms of spike rate at threshold, and one other cluster was separable (*p* < 0.05) in terms of resting membrane potential.

### Biophysical diversity reproduces IP diversity in computational cell models

The above analyses strongly suggested a broad continuum of intrinsic ACC principal cell properties. To understand how such a situation may influence local network behavior, we first modeled this diversity computationally. A set of biophysical computational ACC cell models was generated to capture the above spread of IP values. Cells in this model could reproduce the firing properties of cells observed experimentally (Fig. 4*A*). A range of ion channel conductances was identified for each of eight different ion channels that reproduced the distribution of IP values recorded experimentally (Fig. 4*B*). Notably, this method did not explicitly constrain IP distribution shapes, yet the simulated IPs distributed similarly to the experimental IPs in most cases (Fig. 4*C*). Five IPs explained >85% of the total variance in the experimental data (IP5, AHP duration; IP6, spike width; IP7, spike rate at threshold; IP8, resting membrane potential; and IP9, instantaneous spike frequency) and were included in the model. In addition, we compared the correlations between *z*-scored IP values recorded in each cell with those from all model cells. This analysis showed that each experimental cell had at least one model cell with a value of *R* ^2^ > 0.85, and 90% of experimental cells had at least one model cell with *R* ^2^ > 0.9, indicating a very high correlation between the experimental and modeled IP values.

### β and γ frequency rhythms were generated by different inhibitory decay constants in an ACC network model

To predict a possible role for the observed heterogeneity of IPs, the range of E-cells modeled above were combined with local circuit interneurons and inserted into an ACC network model (Fig. 5). Results from this model were compared with a model containing homogenous E-cell populations in which the intrinsic properties were the same for all cells in the population (see Materials and Methods). Heterogeneity was based on model parameters drawn from a multivariate distribution that preserves the correlation between the biophysical parameters producing cell responses constrained by experimental IPs (see Materials and Methods)

The different β and γ frequencies observed experimentally could be replicated in both the heterogeneous and homogeneous E-cell–containing models by switching the interneuron population inhibitory decay time constant (τ) from 5 to 13 ms (Fig. 5), consistent with experimentally observed values (see above). Simulation of all the heterogeneous E-cell models resulted in a broad distribution of oscillation frequencies, predominantly within either the β- or γ-frequency band, depending on the set inhibitory decay time constant (Fig. 5). This effect was similar regardless of whether the E/I assembly was driven by background activity (Poisson noise) or a rhythmic input. In both cases, cell diversity broadened the range of frequencies generated by the networks, but with different inhibition time constants resulting in largely separable frequency ranges at β and γ frequency (Fig. 5*C*, *D*).

### Network heterogeneity decreases competition and increases synchrony among multiple assemblies

The above simulations led us to hypothesize that the experimentally observed heterogeneity in ACC might confer a computational advantage to a region that may have to combine multiple inputs at different peak frequencies within a given EEG band. To compare the effects of two different inputs on both the homogeneous and heterogeneous E-cell networks, we ran simulations with two E-cell assemblies connected to the same I-cells both receiving external rhythmic inputs (Fig. 6*A*). With this model configuration, we then assessed whether heterogeneity of cell properties in the model altered the network’s response to multiple different inputs. Competition and synchrony were compared between the networks with homogeneous and heterogeneous E-cell assemblies with a shared pool of inhibitory interneurons (I-cells) and τ*_I_* = 5 and 13 ms (Fig. 6*A–Ei*). Fig. 6*B* shows example raster plots for two assemblies driven by rhythmic inputs at 20 and 50 Hz. In the homogeneous network, assembly E2, driven by an input at 20 Hz, dominated overall activity, although assembly E1 was being driven by an input with faster 50-Hz modulation across the population. When spiking occurred in the less active assembly (E1), it had a moderate degree of synchrony with the dominant assembly (E2). In contrast, in the heterogeneous network, receiving the same 20- and 50-Hz inputs, both assemblies were now able to sustain more equal activity levels throughout the simulation, and with a greater degree of overlap in spike timing. Very similar results were obtained with interneuron population inhibitory decay time constants at both τ*_I_* = 5 ms and τ*_I_* = 13 ms. These examples emphasize how a wider diversity of cell properties within assemblies can increase the spike synchrony and decrease competition among multiple assemblies. Over a range of input frequencies *f*_1_ and *f*_2_, the degrees of competition and synchrony between target assemblies E1 and E2 were related to the proximity of their input frequencies. Competition in the heterogeneous network was reduced across all values of *f*_1_ and *f*_2_. Furthermore, for assemblies driven by inputs separated by >30 Hz (i.e., across EEG β- and γ-frequency bands), heterogeneity significantly increased spike synchrony.

Similarly, in separate simulations where only one cell assembly (E1) received an external rhythmic input and the other assembly (E2) received an equal-rate Poisson noise, the degree of competition and synchrony between target assemblies E1 and E2 were related to the frequency *f*_1_ of the external rhythm (Fig. 6*A–Eii*). However, in this condition the interaction involved E1 following an external rhythm and E2 exhibiting a noise-driven local rhythm at its natural frequency (as in Fig. 5*A*). Given this interaction between external and local rhythms, heterogeneity reduced competition across all values of *f*_1_ to a greater extent than occurred for two assemblies driven by external rhythms. Furthermore, a wider diversity of cell properties increased spike synchrony between externally driven and locally generated rhythmic assemblies to a greater extent for β and γ rhythmic inputs. Again, very similar results were obtained with interneuron population inhibitory decay time constants at both τ*_I_* = 5 ms and τ*_I_* = 13 ms (Fig. 6*A–Eii*). Replotting the data as *f*_1_ versus *f*_2_ along separate axes for both τ*_I_* = 5 ms and τ*_I_* = 13 ms shows the largest reduction in competition and increase in synchrony within the β- and γ-frequency bands (Fig. 6*F*, *G*).

## Discussion

The present findings support the evidence that ACC generates γ- and β-frequency oscillations as a consequence of local circuit interactions between principal cells and interneurons. This type of local circuit behavior is near-ubiquitous in cortex (Whittington et al., 2011). The generation of β- and γ-frequency activity does not, alone, therefore present any clues as to the proposed hub-like role of ACC in combining multiple inputs required for its general role in cognitive control (Lapish et al., 2008; Durstewitz et al., 2010; Shenhav et al., 2013; Ma et al., 2014). However, in ACC, we found that this fundamental, inhibition-based mechanism of rhythm generation was present, along with considerable heterogeneity of principal cell intrinsic properties. Computational modeling predicted that an inhibition-based oscillation, combined with such heterogeneity, would have a limited effect on the locally generated rhythm, but a potent effect on the network’s response to diverse oscillatory inputs. Neuronal response heterogeneity caused a transition from a network behavior, in which frequency-selected single inputs generated a single local ACC network output, to a combinatorial behavior, in which the network could combine oscillating inputs of different frequency.

### Local generation of γ and β oscillations

We have demonstrated that γ- and β-frequency oscillations can be evoked in the ACC *in vitro* with application of KA alone. This is consistent with data *in vitro* from the hippocampus (Hájos et al., 2000; Hormuzdi et al., 2001; Fisahn, 2005) and neocortex (Roopun et al., 2008a; Anver et al., 2011; Ainsworth et al., 2012), where KA application has also been shown to evoke fast network oscillations in the 20- to 80-Hz frequency range. Network oscillations in the β- and γ-frequency range in ACC are dependent on GABA_A_ and AMPA receptors (Steullet et al., 2014). With the exception of β rhythms in parietal association areas (Roopun et al., 2006), this pharmacological profile is consistent with other local cortical γ and β oscillations that are an emergent property of the network and reflect the activation by KA of a reciprocally connected pyramidal-fast spiking interneuron network (Whittington et al., 2011).

The distinction between γ- and β-frequency oscillations corresponded to the presence of IPSPs with different decay kinetics recorded from morphologically unidentified cells in ACC. The IPSP values obtained were consistent with the kinetics of GABA_A_ receptor–mediated events associated with γ oscillations in hippocampus and neocortex and β oscillations in auditory cortex (Whittington et al., 1995; Ainsworth et al., 2011). The most parsimonious explanation for these two different frequencies of network activity, and two different inhibitory decay times, would be that distinct interneuron subtypes differentially contributed to the β- and γ-frequency oscillations (Roopun et al., 2008a). PV- and somatostatin-expressing interneurons in the PFC have been shown to contribute to distinct behavioral functions (Kvitsiani et al., 2013; Pinto and Dan, 2015). Such interneuron subtype–specific functions might therefore correlate with the distinct network activities at β and γ frequencies. β-frequency oscillations have been proposed to play a role in establishing functional long-range connections, whereas γ-frequency oscillations are thought to be more important for local interactions (Donner and Siegel, 2011; Kopell et al., 2010). In addition, γ-frequency activity may mediate feed-forward interactions, whereas β-frequency activity has been proposed to mediate feedback interactions (Bastos et al., 2012, 2015; but see below).

### Variability of oscillatory inputs to ACC

A principal underlying the role of oscillations in determining functional connectivity between brain areas is that, within a classic EEG frequency band, they provide a mechanism by which neurons generate outputs at times appropriate for optimizing their mutual influence (Ainsworth et al., 2012). For this so-called communication through coherence to occur, matching the phase and frequency of oscillations in the connected areas is important (Fries, 2005). However, even within a classic EEG band, the network oscillation frequencies can vary enormously. In the case of γ oscillations, frequency can vary as much as 20 Hz depending on the region of origin (Middleton et al., 2008; Herrmann et al., 2010) and the properties of the sensory input that generates them (Orekhova et al., 2015; Perry et al., 2015). Similarly, β oscillations in different brain regions may vary in peak frequency by up to 10 Hz (e.g., van Burik et al., 1998; Roopun et al., 2008b).

Within brain regions receiving concurrent oscillating inputs in the γ or β EEG bands, even subtle frequency differences have been predicted to have dramatic effects. In networks where the dominant time-constant governing rhythmicity is that of synaptic inhibition, one input at a slightly faster frequency than another can effectively abolish any influence the slower frequency has on local spike generation (Cannon et al., 2014). Similarly, synchronous inputs can be readily separated from asynchronous inputs (Akam and Kullmann, 2010), but if multiple inputs arrive at similar frequencies they can become mutually distracting (Akam and Kullmann, 2014). Therefore, if a local network such as the ACC is to combine multiple oscillating inputs, a network property additional to the rhythmicity afforded by local inhibition must be present. Data and computational modeling presented here strongly suggest that heterogeneity in intrinsic neuronal electrophysiological properties may provide such a combinatorial advantage to the network.

### Intrinsic electrophysiological properties of neurons in ACC

Neurons recorded in this study could be subjectively divided into five broad groups similar to those described in other PFC regions (Yang et al., 1996; Dembrow et al., 2010; Gee et al., 2012; Lee et al., 2014; Glykos et al., 2015; van Aerde and Feldmeyer, 2015). However, using a range of established clustering algorithms that have been used successfully in other cortical areas to identify distinct neuronal clusters based on electrophysiological properties (Sosulina et al., 2006; Keshavarzi et al., 2014; Ferrante et al., 2016), we did not identify distinct clusters. Three significantly different clusters could be distinguished using *k*-means clustering, but the clusters were not well separated and they could not be replicated using hierarchical clustering. Our data, therefore, suggest a broad continuum of electrophysiological properties present in ACC neurons, with this heterogeneity mediated by the relative density of intrinsic conductances, including passive and voltage-gated potassium channels, persistent sodium channels, and HCN channels. These channels have overt effects on intrinsic neuronal properties (He et al., 2014) and are vital for controlling the resonance and thus dynamic input-filtering properties of neurons (Hutcheon and Yarom, 2000). In turn, bandpass input filtering is vital for input selection and routing of oscillatory inputs (Kopell et al., 2010; Akam and Kullmann, 2010, 2014).

Neocortical areas can also exhibit considerable heterogeneity in electrophysiological properties as evidenced by the recent extensive documentation of neuronal circuits within the somatosensory cortex (Markram et al., 2015). As discussed by Markram et al. (2015), in addition to intrinsic properties, there can also be heterogeneity of synaptic properties such as decay times, synaptic depression, and facilitation, which can vary between cell types (Thomson et al., 1996). However, Markram et al. (2015) describe electrically stimulated and spontaneous activity in the neocortex, and not the properties of neurons during an emergent network rhythm, such as β- and γ-frequency activity. Our data demonstrate that there is little variability in, for example, decay times of the IPSPs that correlated with either the β- or γ-frequency field oscillation.

One interesting feature we observed was the lack of any clear laminar segregation in the generation of either β- or γ-frequency activity within the ACC. This is in marked contrast to other neocortical areas where several studies *in vitro* have shown that γ-frequency activity is generated in the superficial layers (II/III), whereas β-frequency activity arises from deep (V/VI) layers (Roopun et al., 2008a; Ainsworth et al., 2012). No such obvious laminar distinction was seen in this study, and both β- and γ-frequency activity could be recorded from all layers. This difference in organization may reflect the absence of a functional layer IV in ACC, or may in fact reflect the integrative function of ACC, such that oscillations of different frequencies occur across all the laminae.

### Consequences for ACC functionality in a dynamic network

The data and model presented here suggested that the modal peak frequency of an oscillation in ACC was predominantly governed by the time course of synaptic inhibition. This was the case both when the observed diversity of intrinsic properties was used and when principal neuron properties were homogeneous. The biggest difference made by the observed intrinsic heterogeneity was a broadening of the input-filtering characteristics of the model ACC network and a resulting ability of the network to respond to multiple inputs of differing frequencies concurrently within either the γ or β EEG bands. This is consistent with the ideas of Seamans and colleagues (Lapish et al., 2008; Durstewitz et al., 2010; Ma et al., 2014), who consider ACC as consisting of “overlapping cell assemblies encoding various cognitive events involved in a decision-making process.” The emergence of a broader range of local and resonant frequencies, afforded by cellular diversity, would promote diverse inputs to equally influence the same target (ACC).

Interestingly, with the degree of model heterogeneity constrained by the biological data gathered, the broadening of input filter characteristics predicted a decrease in competition and increased synchrony between ACC regions receiving or generating both γ and β frequencies. This seems antagonistic to proposed functions of discrete frequency bands such as hierarchical organization of signals (Lakatos et al., 2005), concatenation (Roopun et al., 2008b), and segregation of top-down and bottom-up signals (Bastos et al., 2015). However, with ACC sitting at the top of the functional connectivity hierarchy in the cortex, these rules need not apply—e.g., there is no overt top-down input to ACC. In addition, broader filter characteristics were predicted to powerfully reduce competition and increase synchrony when comparing a discrete input frequency with noise alone. This may be seen as a negative property, allowing ACC to generate associations where there are none [e.g. when they are “cognitively false” (Straube et al., 2011)]. However, it may also underlie the observed role of ACC in the formation of novel intuitive associations (Jung-Beeman et al., 2004; Mai et al., 2004).

## Conclusions

A dynamic approach to understanding functional brain connectivity has previously shown that information held in neuronal oscillations can be selected and routed through the cortex on the basis of frequency- and phase-related competition. Here we present an additional dynamic process whereby different frequencies of oscillation can be combined together. The phenomenon is facilitated by the interplay between the kinetics of fast synaptic inhibition (which sets the center frequency for a given oscillation band) and the degree of heterogeneity in intrinsic electrophysiological properties of principal cells (which sets the bandwidth). Although this study has focused on ACC, it is possible that similar mechanisms could be used in other hub regions, where diverse inputs are integrated. A synergistic interplay between synaptic inhibition and intrinsic electrophysiology would provide a rich functional network structure that would be highly labile to neuromodulatory substances such as acetylcholine, dopamine, and noradrenaline (Carr et al., 2007; Hasselmo and Sarter, 2011; Gee et al., 2012; Dembrow and Johnston, 2014). We suggest that neuromodulators may modulate the degree of hub-like or input-selective functionality of higher brain structures to influence cognitive functions (Deco and Rolls, 2003).

## Synthesis

Reviewing Editor: Marlene Bartos, University of Freiburg Decisions are customarily a result of the Reviewing Editor and the peer reviewers coming together and discussing their recommendations until a consensus is reached. When revisions are invited, a fact-based synthesis statement explaining their decision and outlining what is needed to prepare a revision will be listed below. The following reviewer(s) agreed to reveal their identity: Vikaas Sohal, Andreas Draguhn. Note: If this manuscript was transferred from JNeurosci and a decision was made to accept the manuscript without peer review, a brief statement to this effect will instead be what is listed below.

The manuscript describes mechanisms which may facilitate the ability of neural networks in the ACC to integrate information from multiple sources. The authors show that heterogeneity of intrinsic properties of principal neurons supports the integration of different neuronal network rhythms and reduces the competition between sub-networks or cells assemblies.

Reviewer 1:

Based on my previous review, I feel this manuscript is suitable for eNeuro.

Reviewer 2:

1. Results, Local network inhibition (Lines 406 f and Fig. 1): IPSPs were recorded from 10 cells which are not classified. Amongst those the authors find beta-followers, beta-non-followers, and three different cases for mixed beta-gamma field oscillations. This makes up 5 different combinations of field and intracellular rhythms, based on 10 cells (if I did not misunderstand the respective Results section). Given that the 10 neurons might represent different cell types in different layers, this reviewer wonders whether the heterogeneous cell-field coupling can be described in any valid way based on these numbers.

2. Classification of ACC cells according to intrinsic properties (Results, line 463-514): The major part of this section (and accompanying plots in Fig. 2) relies on a 'manual' classification based on rather subjective criteria which lack sharp, quantitative definition. Actually, the whole section reads like a very subjective inspection of a heavily variable data set. The last paragraph then states that two different pattern segregation algorithms failed to detect any clusters. Is it then justified to make a figure with examples and several bar plots based on the 5 categories which, in fact, have no quantitatively defined origin and are nowhere claimed to form real classes? In this reviewer's view, it would be more adequate to show the large variety and -if possible- plots documenting the lack of clearly segregated clusters. Likewise, the respective chapter in Discussion describing the failed approach ('...could be subjectively... However,...') should be omitted.

3. Simulated biophysical diversity (Fig. 3 and respective Results section): The authors reproduce much of the variance of 5 parameters in their simulation (with a significant exception in maximal firing rate which should be stated more explicitly). It does not become clear to this reviewer, however, whether the combinations of parameters in individual cells were comparable between model and measured data. This could be tested, for example, by comparing pairwise covariances in the experimental and simulated data.

4. The main results concern bandwidth of both oscillation patterns (Fig. 4), competition between different rhythms and cell synchrony (Fig. 5). In an accompanying review and rebuttle letter, the reviewer asks for a broader analysis of these interactions, e.g. by resolving interactions between competing networks/ensembles by resolving frequencies f1 and f2 along two axes. He/she also suggests working with two concomitant interneuron populations, thereby modelling the heterogeneity of IPSP frequencies found in recordings.

5. The authors might quote the recent, rigid analysis of neuronal heterogeneity by Markram and colleagues (Markra et al., Cell 2015). It should then critically discussed, whether heterogeneity in the ACC is really larger than in other neocortical networks. This may be important for the interpretation of data with respect to the supposed 'hub' function of the ACC which is, in this reviewer's view, discussed a bit loosely and speculatively.

6. In the discussion, it should also be emphasized that heterogeneity of synaptic interactions with respect to time constants, synaptic strength, failure rates etc. may provide important additional/alternative mechanisms underlying heterogeneity of collective behavior.

Minor:

- Methods, line 220: Data were only processed up to a frequency of 80 Hz.

- Line 303 f: The definition of assemblies is not described in sufficient detail.

- Methods (Recording techniques, line 201 f) and Results (1st paragraph): The authors report no differences in field potentials between layers. The position(s) of recording electrodes and numbers of comparative observations within single slices should be stated to be able to validate this statement. Different oscillation frequencies between layers have been observed in the past, not at last by the same authors.

- Fig. 2 is quoted in the Methods section, before Fig. 1.

- Line 204: please indicate concentration of K-acetate

- Line 234: I suggest to phrase parameter 2 more closely to the raw data, i.e. as amplitude of the (Ih-mediated) sag potential (voltage!). Of course, it is an indirect measure of the underlying current, but this applies to all voltage deflections.

- Line 237-239: what is the 'magnitude' of a potential, integrated over 200 ms? Is it simply the maximal amplitude of the AHP within a window of 200 ms after AP?

- Lines 328/329: typo / grammar error

- Line 1045: Fig. B as a whole is described AFTER panels Bi etc.
